# Bayesian Inference of the Weibull Model Based on Interval-Censored Survival Data

**DOI:** 10.1155/2013/849520

**Published:** 2013-01-10

**Authors:** Chris Bambey Guure, Noor Akma Ibrahim, Mohd Bakri Adam

**Affiliations:** ^1^Institute for Mathematical Research, Universiti Putra Malaysia, 43400 Serdang, Selangor, Malaysia; ^2^Bolgatanga Senior High School, P.O. Box 176, Upper East Region, Bolgatanga, Ghana; ^3^Institute for Mathematical Research and Department of Mathematics, Universiti Putra Malaysia, 43400 Serdang, Salangor, Malaysia

## Abstract

Interval-censored data consist of adjacent inspection times that surround an unknown failure time. We have in this paper reviewed the classical approach which is maximum likelihood in estimating the Weibull parameters with interval-censored data. We have also considered the Bayesian approach in estimating the Weibull parameters with interval-censored data under three loss functions. This study became necessary because of the limited discussion in the literature, if at all, with regard to estimating the Weibull parameters with interval-censored data using Bayesian. A simulation study is carried out to compare the performances of the methods. A real data application is also illustrated. It has been observed from the study that the Bayesian estimator is preferred to the classical maximum likelihood estimator for both the scale and shape parameters.

## 1. Introduction

 One of the features of survival data is censoring. The common one is right censoring and literature on it is well established. Among them are, Al-Aboud [1], Al-Athari [2], Syuan-Rong and Shuo-Jye [3], Guure and Ibrahim [4], Pandey et al. [5], Soliman et al. [6], Abdel-Wahid and Winterbottom [7], Guure et al. [8] and many others. The focus of this study is on interval censoring, which presumably is more demanding than right censoring and, as a result, the approach developed for right censoring does not generally apply.

Interval censoring has to do with a study subject of interest that is not under regular observation. As a result, it is not always possible to observe the failure or survival time of the subject. With interval censoring, one only knows a range, that is, an interval, inside of which one can say the survival event has occurred. Left- or right-censored failure times are special cases of interval-censored failure times. As stated by Turnbull [9], one could define an interval-censored observation as a union of several nonoverlapping windows or intervals.

According to Jianguo [10], interval-censored failure time data occur in many areas including demographical, epidemiological, financial, medical, sociological, and engineering studies. A typical example of interval-censored data occurs in medical or health studies that entail periodic followups, and many clinical trials and longitudinal studies fall into this category. In such situations, interval-censored data may arise in several ways. For instance, an individual may miss one or more observation times that have been scheduled to clinically observe possible changes in disease status and then return with a changed status as stated by Jianguo [10].

Consider individuals who visit clinical centres at times convenient to them rather than the predetermined observation time; in this type of situation, the data obtained are interval-censored. Should all study subjects or units follow the predetermined observation schedule time exactly, it is still not possible to observe the exact time of the occurrence of the change, even when we assume it is a continuous variable. In cases of this nature, one has grouped failure time data, that is, interval-censored data of which the observation for each subject is a member of a collection of non-overlapping intervals.

Researchers who have discussed interval censored data in the classical point of view are, Lawless [11], Flygare and Buckwalter [12], Lindsey [13], Scallan [14], and Odell et al. [15]. To the best of our knowledge, none in the literature so far has interval-censored data using the Bayesian estimation approach with regards to Weibull distribution, which is the essence of this study.

One of the primary advantages of Weibull analysis is its ability to provide reasonably accurate analysis and forecasts with extremely small samples, Abernethy [16]. Small samples allow cost effectiveness.

When the Weibull distribution shape parameter is say, *β* < 1, it indicates that the hazard rate decreases over time implying infant mortality. When *β* = 1, it indicates constant hazard rate over time and *β* > 1 implies the hazard rate increases with time which is as a result of ageing. In summary, the Weibull shape parameter gives the physics behind the death of a biological system while the scale parameter *α* determines the duration of the disease on a biological system. The scale parameter is also referred to as the characteristic life of the distribution.

Even though nonparametric estimation is more widely used in analysing survival data, it is still necessary to discuss parametric estimations. In parametric estimations, the distribution of the survival data is most often assumed to be known. Distributions that are often used in survival analysis are Weibull, exponential, log-logistic, and log-normal. As discussed above, we have assumed that the survival data follow Weibull distribution. The approach used in this paper can be extended to other lifetime distributions.

The paper is structured as follows: Section 2 contains the derivative of the parameters under maximum likelihood estimator, Section 3 is the Bayesian inference. Simulation study is in Section 4 followed by results and discussion in Section 5.  Section 6 is real data analysis and then a conclusion is provided in Section 7.

## 2. Maximum Likelihood Estimation

Let *t*_1_,…, *t*_*n*_ be the lifetimes from a random sample of size *n* where the probability density function (pdf) is represented by *f*(*t*, *α*, *β*), the cumulative distribution function (cdf) is *F*(*t*, *α*, *β*), and the survival function is *S*(*t*, *α*, *β*); given the two-parameter Weibull distribution we have, respectively,

(1) $$\begin{array}{c} f\left(t,\alpha ,\beta \right)=\frac{\beta }{\alpha }{\left(\frac{t}{\alpha }\right)}^{\beta -1}\exp\left[-{\left(\frac{t}{\alpha }\right)}^{\beta }\right], \end{array}$$

(2) $$\begin{array}{c} F\left(t,\alpha ,\beta \right)=1-\exp\left[-{\left(\frac{t}{\alpha }\right)}^{\beta }\right], \end{array}$$

(3) $$\begin{array}{c} S\left(t,\alpha ,\beta \right)=\exp\left[-{\left(\frac{t}{\alpha }\right)}^{\beta }\right], \end{array}$$

where *β* represents the shape parameter and *α* the scale parameter.

Let [*L*_*i*_, *R*_*i*_] denote the interval-censored data and let *T* represent the unknown time, that is, *L*_*i*_ ≤ *T*_*i*_ ≤ *R*_*i*_, where *L*_*i*_ is the last inspection time and *R*_*i*_ the state end time.

If censoring occurs noninformatively and if the law governing *L* and *R* does not involve any of the parameters of interest, we can base our inferences on the likelihood function *L*(*α*, *β*; *L*_*i*_, *R*_*i*_) as stated by Gómez et al. [17], which is given by

(4) L(Li,Ri ∣ α,β)=∏i=1n[F(Ri,α,β)−F(Li,α,β)]=∏i=1n[S(Li)−S(Ri)]=∏i=1nprob{Li≤Ti≤Ri}.

By using (3), we have

(5) $$\begin{array}{c} L\left(L_{i},R_{i}\;|\;\alpha ,\beta \right)=\prod_{i=1}^{n}\left\{\exp\left[-{\left(\frac{L_{i}}{\alpha }\right)}^{\beta }\right]-\exp\left[-{\left(\frac{R_{i}}{\alpha }\right)}^{\beta }\right]\right\}. \end{array}$$

Taking the natural log of (5), we have

(6) $$\begin{array}{c} \ell =\sum_{i=1}^{n}\ln\left\{\exp\left[-{\left(\frac{L_{i}}{\alpha }\right)}^{\beta }\right]-\exp\left[-{\left(\frac{R_{i}}{\alpha }\right)}^{\beta }\right]\right\}. \end{array}$$

To find the values of *α* and *β* that maximize (6), we differentiate (6) with respect to *α* and *β* and set the resulting equations to zero.

Therefore

(7) ∂ℓ∂β =∑i=1n{[−((Liα)βln(Liα)exp[−(Liα)β]        −(Riα)βln(Riα)exp[−(Riα)β])     ×({exp[−(Liα)β]−exp[−(Riα)β]})−1]}=0,∂ℓ∂α =∑i=1n{[−((Liα)β(Liα)exp[−(Liα)β]        −(Riα)β(Riα)exp[−(Riα)β])     ×({exp[−(Liα)β]−exp[−(Riα)β]})−1]}=0.

With a simple numerical approximation, the maximum likelihood estimates of *α* and *β* can be obtained.

## 3. Bayesian Inference

Bayesian inference is an approach that employs the Bayes' rule in order to update the probability estimate of a hypothesis taking into consideration new evidence as it becomes available. Bayesian updating is one of the essential techniques used in modern statistics, more importantly in mathematical statistics. Bayesian updating is particularly important in analysing data that is progressive. Bayesian inference can be applied in many other fields like engineering, medicine, and accounting. The Bayes approach makes use of our prior beliefs of the parameters which is referred to as Prior distribution. The prior is a distribution of the parameters before any data is observed and is given as *p*(*θ*). It also takes into consideration the observed data which is obtained by making use of the likelihood function, and given as *L*(*t* | *θ*).

The Bayes estimator is considered under three loss functions which are important in Bayesian estimations. They are asymmetric (LINEX and general entropy) loss functions and symmetric (squared error) loss function. 

### 3.1. Prior

Prior distribution of the unknown parameters need to be assumed for the Bayesian inference. As discussed by Berger and Sun [18] and subsequently used by Banerjee and Kundu [19], we let *α* take on a Gamma(*a*, *b*) prior with *a* > 0 and *b* > 0. We assume that the prior of *β* is independent of the prior of *α* and is in the neighbourhood of (0, *∞*). Let *v*(*β*) represent the prior of *β*, where

(8) $$\begin{array}{c} \text{Gamma}\left(a,b\right)\propto \alpha^{a-1}\exp\left(-\alpha b\right). \end{array}$$

A joint density function of the data *α* and *β* for interval censored data can be obtained as below

(9) π(α,β,Li,Ri)∝v(β)αa−1exp(−αb) ×∏i=1n{exp[−(Liα)β]−exp[−(Riα)β]}.

Bayesian inference is based on the posterior distribution which is simply the ratio of the joint density function to the marginal distribution function.

The posterior density function of *α* and *β* given the data is

(10) $$\begin{array}{c} \pi^{*}\left({\alpha ,\beta \;|\;L}_{i},R_{i}\right)=\frac{\pi \left(\alpha ,\beta ,L_{i},R_{i}\right)}{\int_{0}^{\infty }\int_{0}^{\infty }\pi \left(\alpha ,\beta ,L_{i},R_{i}\right)d\alpha \;d\beta }. \end{array}$$

### 3.2. The Loss Functions

Having taking into account our prior information which is essential, the Bayes estimator shall be considered under three loss functions. The first loss function under consideration is squared error loss function (SELF) which is symmetrical in nature. What is worth noting is that many estimation problems that involve either one or more parameters are treated in most cases using the symmetric loss function. This loss function gives equal weight to estimation errors that are the same regardless of whether the loss obtained has either overestimated or underestimated the parameter or the problem being investigated. At some particular times we observe that estimation errors with respect to a particular problem are preferable either in one direction or the other. When one takes into perspective a univariate problem, it can be stated that an overestimation may cause more seriousness than that of an underestimation or vice versa as also stressed by Hamada et al. [20]. Varian [21] was motivated by the use of asymmetric loss functions and therefore applied it in estimating problems arising in real estate assessment, where overestimation of a property's value might cause it to remain on the market unsold for an extended period, ultimately costing the seller superfluous or gratuitous expenses. As a result of the above discussions, we have taken into perspective two asymmetric loss functions, which are linear exponential (LINEX) loss function and general entropy loss function (GELF).

(1) The squared error loss function is given as
  (11) $$\begin{array}{c} L\left(a,\theta \right)={\left(a-\theta \right)}^{2}, \end{array}$$
  where *L*(*a*, *θ*) is the loss incurred by adopting action *a* when the true state of nature is *θ*.
(2) The LINEX loss function is also given as
  (12) $$\begin{array}{c} L\left(\Delta \right)=e^{c\Delta }-c\Delta -1,\;c\neq 0, \end{array}$$
  where $\Delta =(\hat{\theta }-\theta )$, with $\hat{\theta }$ being the estimate of *θ*. According to Zellner [22], the posterior expectation of the LINEX loss function is
  (13) $$\begin{array}{c} E_{\theta }\left[L\left(\Delta \right)\right]=e^{c\hat{\theta }}E_{\theta }e^{-c\theta }-c\left(\hat{\theta }-E_{\theta }\theta \right)-1. \end{array}$$
  The value of $\hat{\theta }$ that minimizes the above equation is
  (14) $$\begin{array}{c} {\hat{\theta }}_{\text{BL}}=-\frac{1}{c}\ln E_{\theta }\left(e^{-c\theta }\right) \end{array}$$
  provided *E*_*θ*_(·) exists and is finite.
(3) Another useful asymmetric loss function is the general entropy (GELF) which is a generalization of the entropy loss and is given as
  (15) $$\begin{array}{c} L\left(\hat{\theta }-\theta \right)\propto {\left(\frac{\hat{\theta }}{\theta }\right)}^{k}-k\ln\left(\frac{\hat{\theta }}{\theta }\right)-1. \end{array}$$
  The Bayes estimator ${\hat{\theta }}_{\text{BG}}$ of *θ* under the general entropy loss is
  (16) $$\begin{array}{c} {\hat{\theta }}_{\text{BG}}={\left[E_{\theta }(\theta^{-k})\right]}^{-1/k}, \end{array}$$
  provided *E*_*θ*_(·) exists and is finite.

It may be noted that (10) contains double integrals which cannot be solved analytically; this is due to the complex form of the likelihood function given in (5). Therefore, we propose to use Lindley approximation method to evaluate the integrals involved.

### 3.3. Lindley Approximation

 A prior of *β* needs to be specified here so as to calculate the approximate Bayes estimates of *α* and *β*. Having specified a prior for *α* as Gamma(*a*, *b*), it is similarly assumed that *v*(*β*) also takes on a Gamma(*c*, *d*) prior.

According to Lye et al. [23], the posterior Bayes estimator of an arbitrary function *u*(*θ*) given by Lindley is

(17) $$\begin{array}{c} E\left\{u\left(\theta \right)\;|\;x\right\}=\frac{\int \omega \left(\theta \right)\exp\left[\ell \left(\theta \right)\right]d\theta }{\int v\left(\theta \right)\exp\left[\ell \left(\theta \right)\right]d\theta }, \end{array}$$

where *ℓ*(*θ*) is the log likelihood and *ω*(*θ*), *v*(*θ*) are arbitrary functions of *θ*. We assume that *v*(*θ*) is the prior distribution for *θ* and *ω*(*θ*) = *u*(*θ*) · *v*(*θ*) with *u*(*θ*) being some function of interest. 

Equation (17) can be approximated asymptotically by the following:

(18) E{u(θ) ∣ x}=[u+12∑i∑j(uij+2ui·ρj)·σij           +12∑i∑j∑k∑lℓijk·σij·σkl·ul],

where *i*, *j*, *k*, *l* = 1,2,…, *n*;  *θ* = (*θ*_1_, *θ*_2_,…, *θ*_*m*_). 

Taking the two parameters into consideration, (18) reduces to

(19) θ^=u+12[(u11σ11)+(u22σ22)]+u1ρ1σ11  +u2ρ2σ22+12[(ℓ30u1σ112)+(ℓ03u2σ222)],

where *ℓ* is the log-likelihood function in (6). If *u*(*α*, *β*) is some function of interest, then under squared error loss function we have

(20) π∗(α,β ∣ Li,Ri)=∬u(α,β)π(α,β,Li,Ri)dα dβ∬π(α,β,Li,Ri)dα dβ,ρ=lnv1(α)+lnv(β),ρ1=c−1α−d,  ρ2=a−1β−b,u(α)=α,  u1=∂u∂α=1,u11=∂2u∂α2=0,  u(β)=β,  u2=∂u∂β=1,  u22=∂2u∂β2=0.

Let the following definitions hold:

(21) x=exp[−(Liα)β],    y=exp[−(Riα)β],e=[−(Liα)β],    f=[−(Riα)β],w=[−ln(Liα)],    a=[−ln(Riα)],ℓ20=∂2ℓ∂α2=∑i=1n{[1x−y]         ×{e(β2α2)x+e(βα2)x+e2             ×(β2α2)x−f(β2α2)y               −f(βα2)y−f2(β2α2)y}           −{[e(β/α)x+f(β/α)y]2[x−y]2}},σ11=(−ℓ20)−1,   σ22=(−ℓ02)−1,ℓ30=∂3ℓ∂α3=∑i=1n{[1x−y]           ×{−e(β3α3)x−3e(β2α3)x            −3e2(β3α3)x−2f(βα3)x            −3e2(β2α3)x−e3(β3α3)x            +f(β3α3)y+3f(β2α3)y            +3f2(β3α3)y+2f(βα3)y              +3f2(β2α3)y+f3(β3α3)y}         −[1x−y]2         ×{3[e(β2α2)x+e(β2α2)x            +e2(β2α2)x−f(β2α2)y            −f(βα2)y−f2(β2α2)y]            ×[−e(βα)x+f(βα)y]}         +{2[−e(β/α)x+f(β/α)y]3[x−y]3}},ℓ02=∂ℓ2∂β2=[1x−y]×{ew2x+e2w2x−fa2y−f2a2y}  −{[ewx−fay]2[x−y]2},ℓ03=∂ℓ3∂β3=[1x−y]×{ew3x+3e2w3x+e3w3x               −fa3y−3f2a3y−f3a3y}  −[1x−y]2×{3{ew2x+e2w2x            −fa2y−f2a2y}{ewx−fay}}  +{2[ewx−fay]3[x−y]3}.

### 3.4. Linear Exponential Loss Function

The Bayes estimator ${\hat{u}}_{\text{BL}}$ of a function *u* = *u*[exp(−*aα*), exp(−*aβ*)] under the LINEX loss function is given as

(22) π∗(α,β ∣ Li,Ri) =∬u[exp(−cα),exp(−cβ)]π(α,β,Li,Ri)dα dβ∬π(α,β,Li,Ri)dα dβ.

The LINEX loss is obtained by using the same Lindley procedure in (19) with

(23) u(α)=e−cα,    u(β)=e−cβ,u1=−ce−cα,    u11=c2e−cα,u2=−ce−cβ,  u22=c2e−cβ.

### 3.5. General Entropy Loss Function

 The Bayes estimator under this loss function with some function of interest, say, *u* = *u*[(*α*)^−*k*^, (*β*)^−*k*^], is

(24) π∗(α,β ∣ Li,Ri) =∬u[(α)−k,(β)−k]π(α,β,Li,Ri)dα dβ∬π(α,β,Li,Ri)dα dβ.

Similar Lindley approach is used for the general entropy loss function as in the squared error loss but here the Lindley approximation procedure as stated in (19), where *u*_1_,  *u*_11_ and *u*_2_, *u*_22_ are the first and second derivatives for *α* and *β*, respectively, are given as

(25) u=(α)−k,    u1=∂u∂α=−k(α)−k−1,u11=∂2u∂(α)2=−(−k2−k)(α)−k−2,  u2=u22=0,u=(β)−k,  u2=∂u∂β=−k(β)−k−1,u22=∂2u∂(β)2=−(−k2−k)(β)−k−2,    u1=u11=0.

## 4. Simulation Study

A simulation study is carried out to determine the best estimator for the two parameters of the Weibull distribution with interval censoring.

Generating the interval-censored data involved the following steps. Each data set contains 25, 50, and 100 interval censored observations. Some observations were left censored, but we have not specified left-censored data from interval censored data.

We assume that the true survival time follows a Weibull distribution.

(a) Generate from the Weibull distribution, say, *t* of size *n* = 25,50, and 100 with *α* = 2 and 4, and *β* = 0.8 and 1.2 to represent the true survival time with a decreasing and increasing shape parameter.
(b) Generate a vector, say, *V* for a set of clinic visits. Assume there are 5 clinic visits, for the Weibull distribution; take the first visit to be *v*_1_ and generate from *Unif*(0, *b*). The next visit of *v*_2_ is also generated from *Unif*(*v*_1_, *v*_2_ + *b*). Subsequent generations are employed with similar approach.
(c) Generate a set of matrix named bounds for each of the data set. To obtain the lower and upper bounds we made use of the following:
  (26) bounds [i,1]={0,if  t<V[i,1],V[i,j],if  V[i,j]<t<V[i,j+1], where  j=1,2,3,4,V[5],if  t>[5]bounds [i,2]={V[i,1],if  t<V[i,1],V[i,j+1],if  V[i,j]<t<V[i,j+1], where  j=1,2,3,4,1000,if  t>V[5],
(d) An indicator is defined such that
  (27) $$\begin{array}{c} \text{indicator}\left[i\right]=\{\begin{array}{cc} 0, & \text{if}\;\;\text{bounds }\left[i,2\right]=1000,\\ 1, & \text{otherwise.} \end{array} \end{array}$$

The coding and the analysis were performed using the R programming language which is freely available. The parameters were estimated with maximum likelihood and Bayesian. To compute the Bayes estimates, assumptions are made such that *α* and *β* take respectively Gamma(*a*, *b*) and Gamma(*c*, *d*) priors. We set the hyperparameters to zero, that is *a* = *b* = *c* = *d* = 0 in order to obtain noninformative priors. Note that at this point the priors become nonproper but the results do not have any significant difference with the implementation of proper priors as also stated by Benerjee and Kundu [19]. The values for the loss parameters were taken to be *c* = *k* = ±  0.6 and *c* = *k* = ±  1.4. A detailed discussion on the choice of the loss parameter can be seen in Calabria and Pulcini [24]. We iterated the simulation process (R) 1000 times to obtain the estimates of the parameters. The mean squared errors and absolute biases are determined and presented below for the purpose of comparison.

To obtain the MSEs and absolute biases for each estimated value, the MSEs and absolute biases are calculated for each of the one thousand estimated values of the scale parameter and the shape parameter that is from 1 to 1000. At the end, what we obtain is the average of MSEs and absolute biases. Our aim is to find out how close the estimated values of the estimators are to the true values at each level of the simulation taking into account that *r* = 1,2,…, 1000.

## 5. Results and Discussion

Table 1 depicts the values of the mean squared error while Table 2 contains the absolute biases for the scale parameter (*α*). In Table 1, we observed that the most dominant estimator that had the smallest mean squared error is Bayesian with both linear exponential (LINEX) and general entropy loss functions. The LINEX loss function is slightly better than the general entropy loss function but both are better than Bayes using the squared error loss and that of the maximum likelihood estimator. We realised that MLE as compared to Bayesian only has the smallest mean squared error at *n* = 25 and 50 with *α* = 4 and *β* = 0.8, where the value for *β* here represents infant mortality. With Table 2, both LINEX and GELF have equal minimum absolute biases for the scale parameter. Considering the standard errors of the estimators given in Table 5, it is observed that, both LINEX and general entropy loss functions perform quite well since they both have almost equal smallest standard errors for the scale parameter. LINEX loss function turn to overestimate the scale parameter.

Considering Tables 3 and 4, we noticed that Bayesian with the assumed informative prior performed astonishingly well under the general entropy and the linear exponential loss functions than the maximum likelihood estimator and that of Bayes under the squared error loss function. The LINEX loss function performed very well with small sample size whiles the general entropy loss function gives the smallest mean squared error with relatively large sample sizes. The minimum biases for the shape parameter occur predominantly with the general entropy loss function followed by linear exponential loss function. 

What we observed here is that, in estimating the shape parameter of the Weibull model with interval censoring, Bayesian estimator with the general entropy loss function may be preferred to the others when we consider mean squared errors and minimal biases of the estimators. This is followed by Bayesian also with the LINEX loss function. The general entropy and the LINEX loss functions underestimate the shape parameter, this is because all the smallest mean squared errors occur at *k* = −1.4, which is less than zero in the case of the general entropy loss, but for the LINEX loss they occur at *c* = −0.6. Considering the standard errors of the estimators given in Table 6, it is observed that LINEX loss function predominantly performs better than the others having obtained smallest standard errors for the shape parameter.

## 6. Real Data Analysis

 We analyse a data set in this section for illustration and comparison purposes. The data is a retrospective study obtained from Lawless [11]. It was carried out to compare the cosmetic effects of radiotherapy versus radiotherapy and adjuvant chemotherapy on women with early breast cancer.

To compare the two treatments, a retrospective study of 46 radiation only and 48 radiation plus chemotherapy patients were conducted. Patients were observed initially for every 4–6 months, but, as their recovery progressed, the interval between visits lengthened. The event of interest was the time for the first appearance of moderate or severe breast retraction. As the patients were observed only at some random times, the exact time, *T*_*i*_, of breast retraction is known only to fall within the interval between visits.

Patients with no moderate or severe breast retraction until the last visit were classified as right censored and then the end point of their intervals was assumed to be *R*_*i*_ = *∞* and *L*_*i*_ was assumed as the time from the beginning to the last visit. Here, our primary concern is with women who were under the same group, that is, radiotherapy and adjuvant chemotherapy for the purpose of illustration. The data are presented in Table 7.

 As clearly presented in Table 8, the estimator which gives the smallest standard error is Bayesian under linear exponential loss followed by general entropy loss function. This is so because both loss functions take into consideration an overestimation and underestimation, even though they have different approaches. Again, we observe that both linear exponential and general entropy loss functions overestimate the scale and shape parameters of the Weibull distribution, that is, *c* = *k* > 0.

In a practical perspective, the use of symmetric loss function is based on the assumption that the loss is the same in any direction; that is, it is simply the magnitude of the losses incurred. Therefore, if one is not interested in knowing whether the assumption made with regards to the duration or the mortality state of the patients being investigated gives a signal as to whether our conclusion is above or below the actual duration or life expectancy of the patient, then this loss function could be used. However, this assumption may not be valid in many practical situations and the use of the symmetric loss function may be inappropriate. For instance, underestimating the blood pressure of a patient can have a very serious consequences on the patient's treatment than overestimating or vice versa. The gravity of the situation is where one knows that the patient has problem with the blood level but does not know if it is high or low to recommend a proper treatment.

Again from Table 8, it is evident and clear that, at 95% confidence or credible intervals, the estimator with narrower confidence or credible intervals is Bayesian, first with the linear exponential loss function followed by general entropy loss function. Notwithstanding the fact that the Bayesian estimator gives narrower credible intervals as compared to the classical maximum likelihood estimator, there is still an advantage for using the Bayesian credible intervals over the classical confidence intervals. Briefly, a frequentist 95% confidence interval of, say, (2,4) means, with a large number of “repeated” new samples at a time, 95% of the calculated confidence intervals would include the true value of the parameter, this is in contrast to the Bayesian analogy of credible interval, which states that with a small sample “not repeatedly” contains the true parameter 95% at a time. In the Bayesian approach the parameter is observed as being random and sought for. 

The values in bold indicate the smallest standard errors and narrower confidence or credible intervals of the preferred estimator.

## 7. Conclusion

In this study, we have taken into consideration the point estimate of the Weibull distribution parameters based on interval censoring through simulation. MLE and Bayes estimators are used to estimate the scale and shape parameter of this lifetime distribution. The Bayes estimators are obtained using linear exponential, general entropy, and squared error loss functions. We also employed the Bayesian non-informative prior approach in estimating the scale and shape parameter after having considered an informative (Gamma) priors. In order to reduce the complicated integrals that are in the posterior distribution which cannot explicitly be obtained in close form, we employed the Lindley approximation procedure to calculate the Bayes estimators.

The Bayesian estimator performed quite well in estimating the scale parameter with respect to the mean squared errors under LINEX loss function than the other loss functions and that of the classical maximum likelihood estimator. With the shape parameter, the Bayesian estimator performed better than the other estimators with general entropy loss function. This is due to the fact that the loss parameter when considered under general entropy has minimal influence on the posterior distribution, thereby exhibiting little biasedness as compared to the LINEX loss function. With respect to the real data, it is observed that Bayes under LINEX loss function gives the smallest standard errors and narrower credible intervals compared to the others but overestimates both parameters. This is followed closely by general entropy loss function. The standard errors for the simulation study indicate Bayes estimator via LINEX is better than the others for the scale parameter but for the shape parameter both LINEX and general entropy performed quite well in estimating it.

## Figures and Tables

**Table 1:** MSEs for $\left(\hat{\alpha }\right)$ under informative prior and MLE with interval-censoring.

*n*	α	β	${\hat{\alpha }}_{\text{ML}}$	${\hat{\alpha }}_{\text{BS}}$	${\hat{\alpha }}_{\text{BL}}$	${\hat{\alpha }}_{\text{BG}}$	${\hat{\alpha }}_{\text{BL}}$	${\hat{\alpha }}_{\text{BG}}$	${\hat{\alpha }}_{\text{BL}}$	${\hat{\alpha }}_{\text{BG}}$	${\hat{\alpha }}_{\text{BL}}$	${\hat{\alpha }}_{\text{BG}}$
					*c* = *k* = 0.6		*c* = *k* = −0.6		*c* = *k* = 1.4		*c* = *k* = −1.4	
25	2	0.8	0.00203	0.00210	0.00209	0.00211	0.00210	**0.00202**	0.00207	0.00213	**0.00202**	0.00204
		1.2	0.00125	0.00145	0.00129	0.00135	**0.00118**	0.00134	0.00127	0.00128	0.00122	0.00162
	4	0.8	**0.00783**	0.00790	0.00816	0.00857	0.00839	0.00788	0.00826	0.00826	0.00795	0.00801
		1.2	0.00501	0.00633	0.00529	0.00511	0.00556	0.00633	0.00523	0.00548	**0.00489**	0.00606

50	2	0.8	0.00096	0.00096	0.00097	**0.00094**	0.00095	0.00096	0.00097	0.00099	0.00096	0.00098
		1.2	0.00059	0.00058	0.00059	0.00058	0.00064	0.00059	0.00058	**0.00057**	0.00058	0.00058
	4	0.8	**0.00375**	**0.00375**	0.00388	0.00379	0.00392	0.00383	0.00382	0.00386	0.00387	0.00389
		1.2	0.00241	0.00240	0.00237	0.00240	0.00277	0.00235	0.00231	0.00237	**0.00228**	0.00272

100	2	0.8	0.00064	0.00064	0.00062	0.00063	0.00063	**0.00061**	0.00062	0.00063	0.00064	**0.00061**
		1.2	**0.00037**	0.00041	0.00038	0.00039	**0.00037**	**0.00037**	**0.00037**	**0.00037**	0.00047	0.00045
	4	0.8	0.00249	0.00251	0.00253	0.00255	0.00253	0.00248	0.00256	0.00249	**0.00244**	0.00248
		1.2	0.00150	0.00149	0.00149	0.00152	0.00260	0.00149	0.00150	0.00153	**0.00147**	0.00152

ML: Maximum Likelihood, BG: General Entropy Loss Function, BL: LINEX Loss Function, BS: Squared Error Loss Function.

**Table 2:** Absolute biases for $\left(\hat{\alpha }\right)$ under informative prior and MLE with interval-censoring.

*n*	*α*	*β*	${\hat{\alpha }}_{\text{ML}}$	${\hat{\alpha }}_{\text{BS}}$	${\hat{\alpha }}_{\text{BL}}$	${\hat{\alpha }}_{\text{BG}}$	${\hat{\alpha }}_{\text{BL}}$	${\hat{\alpha }}_{\text{BG}}$	${\hat{\alpha }}_{\text{BL}}$	${\hat{\alpha }}_{\text{BG}}$	${\hat{\alpha }}_{\text{BL}}$	${\hat{\alpha }}_{\text{BG}}$
					*c* = *k* = 0.6		*c* = *k* = −0.6		*c* = *k* = 1.4		*c* = *k* = −1.4	
25	2	0.8	**0.00600**	0.00605	0.00609	0.00613	0.00602	**0.00600**	0.00607	0.00614	0.00640	0.00602
		1.2	0.00461	0.00460	0.00471	0.00478	**0.00440**	0.00469	0.00459	0.00464	0.00472	0.00474
	4	0.8	0.01177	**0.01175**	0.01211	0.01234	0.01219	0.01186	0.01212	0.01209	0.01273	0.01189
		1.2	**0.00920**	0.01251	0.00947	0.00934	0.01033	0.00955	0.00941	0.00965	0.01053	0.01102

50	2	0.8	0.00300	0.00300	0.00303	0.00297	0.00299	0.00300	0.00301	0.00305	**0.00296**	0.00303
		1.2	0.00232	0.00230	0.00232	0.00231	0.00229	0.00232	0.00230	**0.00228**	0.00262	0.00248
	4	0.8	0.00595	0.00595	0.00605	0.00598	0.00608	0.00601	0.00599	0.00602	**0.00580**	0.00604
		1.2	0.00469	0.00520	0.00465	0.00469	0.00471	0.00465	0.00459	0.00464	0.00463	**0.00454**

100	2	0.8	0.00202	0.00202	0.00199	0.00201	0.00200	**0.00198**	0.00199	0.00200	0.00200	**0.00198**
		1.2	0.00153	0.00154	0.00155	0.00154	**0.00152**	0.00158	**0.00152**	0.00181	0.00197	0.00154
	4	0.8	0.00399	0.00400	0.00402	0.00404	0.00402	0.00398	0.00404	0.00399	**0.00394**	0.00399
		1.2	0.00307	0.00304	0.00305	0.00309	0.00306	0.01164	0.00307	0.00310	0.00309	**0.00302**

ML: Maximum Likelihood, BG: General Entropy Loss Function, BL: LINEX Loss Function, BS: Squared Error Loss Function.

**Table 3:** MSEs for $\left(\hat{\beta }\right)$ under informative prior and MLE with interval-censoring.

*n*	*α*	*β*	${\hat{\beta }}_{\text{ML}}$	${\hat{\beta }}_{\text{BS}}$	${\hat{\beta }}_{\text{BL}}$	${\hat{\beta }}_{\text{BG}}$	${\hat{\beta }}_{\text{BL}}$	${\hat{\beta }}_{\text{BG}}$	${\hat{\beta }}_{\text{BL}}$	${\hat{\beta }}_{\text{BG}}$	${\hat{\beta }}_{\text{BL}}$	${\hat{\beta }}_{\text{BG}}$
					*c* = *k* = 0.6		*c* = *k* = −0.6		*c* = *k* = 1.4		*c* = *k* = −1.4	
25	2	0.8	0.07429	0.06822	0.07525	0.07488	**0.06076**	0.07060	0.07187	0.07383	0.07329	0.06216
		1.2	0.07955	0.07076	0.08011	0.08018	**0.06258**	0.07467	0.08170	0.08185	0.08246	0.06353
	4	0.8	0.07416	0.06799	0.07488	0.07387	**0.06054**	0.07032	0.07221	0.07403	0.07389	0.06336
		1.2	0.08130	0.07267	0.07853	0.07705	**0.05980**	0.07809	0.08272	0.07985	0.08483	0.06331

50	2	0.8	0.03147	0.03026	0.03166	0.03307	0.02986	0.03186	0.03202	0.03201	0.03282	**0.02961**
		1.2	0.03289	0.03123	0.04675	0.03278	0.03194	0.03298	0.03371	0.03457	0.03434	**0.03072**
	4	0.8	0.03248	0.03123	0.03222	0.03274	0.03005	0.03142	0.03266	0.03248	0.03229	**0.03002**
		1.2	0.03359	0.03188	0.03424	0.03420	0.03125	0.03242	0.03352	0.03423	0.03473	**0.03105**

100	2	0.8	0.02093	0.02041	0.02118	0.02061	0.01987	0.02062	0.02088	0.02057	0.02053	**0.01986**
		1.2	0.02142	0.02073	0.02148	0.02166	0.02062	0.02160	0.02165	0.02205	0.02144	**0.02015**
	4	0.8	0.02052	0.02001	0.02041	0.02055	0.02002	0.02053	0.02051	0.02069	0.02098	**0.01958**
		1.2	0.021534	0.02083	0.02224	0.02121	0.02052	0.02099	0.02166	0.02135	0.02186	**0.01999**

ML: Maximum Likelihood, BG: General Entropy Loss Function, BL: LINEX Loss Function, BS: Squared Error Loss Function.

**Table 4:** Absolute biases for $\left(\hat{\beta }\right)$ under informative prior and MLE with interval-censoring.

*n*	*α*	*β*	${\hat{\beta }}_{\text{ML}}$	${\hat{\beta }}_{\text{BS}}$	${\hat{\beta }}_{\text{BL}}$	${\hat{\beta }}_{\text{BG}}$	${\hat{\beta }}_{\text{BL}}$	${\hat{\beta }}_{\text{BG}}$	${\hat{\beta }}_{\text{BL}}$	${\hat{\beta }}_{\text{BG}}$	${\hat{\beta }}_{\text{BL}}$	${\hat{\beta }}_{\text{BG}}$
					*c* = *k* = 0.6		*c* = *k* = −0.6		*c* = *k* = 1.4		*c* = *k* = −1.4	
25	2	0.8	0.03694	0.03538	0.03713	0.03723	0.03645	0.03612	0.03652	0.03685	0.03699	**0.03392**
		1.2	0.03770	0.03555	0.03795	0.03790	**0.03351**	0.03641	0.03829	0.03822	0.03836	0.03371
	4	0.8	0.03701	0.03544	0.03727	0.03689	0.03367	0.03609	0.03653	0.03699	0.03651	**0.03409**
		1.2	0.03824	0.03612	0.03745	0.03721	0.03287	0.03727	0.03838	0.03775	0.03750	**0.03375**

50	2	0.8	0.01742	0.01708	0.01747	0.01783	0.01698	0.01749	0.01755	0.01756	0.01762	**0.01689**
		1.2	0.01770	0.01724	0.01816	0.01766	0.01710	0.01767	0.01787	0.01807	0.01737	**0.01707**
	4	0.8	0.01767	0.01732	0.01766	0.01776	0.01702	0.01740	0.01772	0.01769	0.01772	**0.01698**
		1.2	0.01783	0.01736	0.01801	0.01802	0.01721	0.01757	0.01784	0.01805	0.01793	**0.01714**

100	2	0.8	0.01166	0.01159	0.01173	0.01158	0.01137	0.01159	0.01166	0.01157	0.01223	**0.01136**
		1.2	0.01176	0.01157	0.01176	0.01180	0.01152	0.01177	0.01181	0.01191	0.01170	**0.01141**
	4	0.8	0.01156	0.01142	0.01153	0.01156	0.01141	0.01156	0.01155	0.01162	0.01167	**0.01128**
		1.2	0.01178	0.01159	0.01197	0.01170	0.01149	0.01164	0.01179	0.01174	0.01172	**0.01135**

ML: Maximum Likelihood, BG: General Entropy Loss Function, BL: LINEX Loss Function, BS: Squared Error Loss Function.

**Table 5:** Standard errors for $\left(\hat{\alpha }\right)$ under informative prior and MLE with interval-censoring.

*n*	*α*	*β*	${\hat{\alpha }}_{\text{ML}}$	${\hat{\alpha }}_{\text{BS}}$	${\hat{\alpha }}_{\text{BL}}$	${\hat{\alpha }}_{\text{BG}}$	${\hat{\alpha }}_{\text{BL}}$	${\hat{\alpha }}_{\text{BG}}$	${\hat{\alpha }}_{\text{BL}}$	${\hat{\alpha }}_{\text{BG}}$	${\hat{\alpha }}_{\text{BL}}$	${\hat{\alpha }}_{\text{BG}}$
					*c* = *k* = 0.6		*c* = *k* = −0.6		*c* = *k* = 1.4		*c* = *k* = −1.4	
25	2	0.8	0.11498	0.11498	0.11087	0.11146	0.11083	0.12784	0.11816	**0.11006**	0.11816	0.12376
		1.2	0.12488	0.12497	0.12950	0.11922	0.12958	**0.11253**	0.12932	0.12659	0.12934	0.11881
	4	0.8	0.24109	0.24109	**0.22301**	0.25215	0.22302	0.24821	0.22862	0.22934	0.22862	0.24554
		1.2	0.22811	0.22813	0.26706	0.24969	0.26716	**0.22134**	0.23055	0.23157	0.23117	0.24681

50	2	0.8	0.08627	0.08627	0.08813	0.08875	0.08814	0.08977	0.08603	**0.08284**	0.08603	0.08609
		1.2	0.08748	0.08789	0.08751	0.08883	0.08752	0.08603	0.08438	0.08471	0.08441	**0.08208**
	4	0.8	0.18219	0.18219	**0.16053**	0.16594	0.16054	0.17512	0.16440	0.16063	0.16440	0.17894
		1.2	0.17697	0.17701	0.18844	0.18104	0.18849	0.18411	**0.17464**	0.18804	0.17471	0.17963

100	2	0.8	0.06947	0.06947	0.06835	0.06723	0.06835	0.07260	**0.06618**	0.07216	0.06618	0.06568
		1.2	0.07325	0.07325	0.07242	0.07056	0.07243	0.07236	**0.06788**	0.06896	0.06790	0.07555
	4	0.8	0.13564	0.13564	0.13236	**0.12725**	0.13237	0.13759	0.13198	0.13536	0.13198	0.14525
		1.2	0.14133	0.14132	0.14946	0.14204	0.14952	0.13963	0.13924	0.15525	**0.13825**	0.14808

ML: Maximum Likelihood, BG: General Entropy Loss Function, BL: LINEX Loss Function, BS: Squared Error Loss Function.

**Table 6:** Standard errors for $\left(\hat{\beta }\right)$ under informative prior and MLE with interval-censoring.

*n*	*α*	*β*	${\hat{\beta }}_{\text{ML}}$	${\hat{\beta }}_{\text{BS}}$	${\hat{\beta }}_{\text{BL}}$	${\hat{\beta }}_{\text{BG}}$	${\hat{\beta }}_{\text{BL}}$	${\hat{\beta }}_{\text{BG}}$	${\hat{\beta }}_{\text{BL}}$	${\hat{\beta }}_{\text{BG}}$	${\hat{\beta }}_{\text{BL}}$	${\hat{\beta }}_{\text{BG}}$
					*c* = *k* = 0.6		*c* = *k* = −0.6		*c* = *k* = 1.4		*c* = *k* = −1.4	
25	2	0.8	0.39065	0.37703	0.32118	0.39273	0.31689	0.43780	0.34444	**0.29605**	0.35674	0.38245
		1.2	0.38779	**0.37700**	0.45495	0.46317	0.46953	0.39625	0.57534	0.43380	0.63824	0.44722
	4	0.8	0.43193	0.42128	0.35109	0.39801	0.35516	0.43867	**0.34216**	0.37998	0.35709	0.41861
		1.2	0.45815	0.44019	0.51752	**0.40865**	0.52893	0.46122	0.58113	0.48520	0.66063	0.44205

50	2	0.8	0.28239	0.27899	**0.25804**	0.34324	0.25969	0.31017	0.25999	0.28062	0.26553	0.28327
		1.2	0.39845	0.39525	0.30510	0.31771	0.30861	0.36466	**0.29946**	0.35294	0.38685	0.31082
	4	0.8	0.29615	0.29352	0.25589	0.30983	0.25788	0.33484	**0.25251**	0.29724	0.25568	0.39005
		1.2	0.35876	0.35389	0.37364	0.36943	0.37841	0.35505	**0.30358**	0.37363	0.30968	0.32348

100	2	0.8	0.24735	0.24534	0.23836	**0.21764**	0.23967	0.25223	0.23995	0.28540	0.24323	0.21909
		1.2	0.29522	0.29258	0.32080	0.27977	0.32392	0.31330	**0.25719**	0.28065	0.26031	0.25282
	4	0.8	0.23126	0.22961	0.26014	0.22817	0.26224	0.22838	**0.20963**	0.25202	0.21198	0.26260
		1.2	0.27372	0.27095	0.26946	**0.25135**	0.27124	0.25875	0.27826	0.31875	0.28261	0.29688

ML: Maximum Likelihood, BG: General Entropy Loss Function, BL: LINEX Loss Function, BS: Squared Error Loss Function.

**Table 7:** Radiotherapy and chemotherapy data.

(8,12]	(0,5]	(30,34]
(0,22]	(5,8]	(13, ∞]
(24,31]	(12,20]	(10,17]
(17,27]	(11, ∞]	(8,21]
(17,23]	(33,40]	(4,9]
(24,30]	(31, ∞]	(11, ∞]
(16,24]	(13,39]	(14,19]
(13, ∞]	(19,32]	(4,8]
(11,13]	(34, ∞]	(34, ∞]
(16,20]	(13, ∞]	(30,36]
(18,25]	(16,24]	(18,24]
(17,26]	(35, ∞]	(16,60]
(32, ∞]	(15,22]	(35,39]
(23, ∞]	(11,17]	(21, ∞]
(44,48]	(22,32]	(11,20]
(14,17]	(10,35]	(48, ∞]

**Table 8:** Standard errors and confidence/credible intervals for $\left(\hat{\alpha }\right)$ and $\left(\hat{\beta }\right)$.

		$\hat{\alpha }$	$\text{s.e }\left(\hat{\alpha }\right)$	$\text{CI }\left(\hat{\alpha }\right)$	$\hat{\beta }$	$\text{s.e }\left(\hat{\beta }\right)$	$\text{CI }\left(\hat{\beta }\right)$
MLE		27.78300	2.00507	(23.8531−31.7129)	2.11394	0.30512	(1.5159−2.7120)
BS		27.77308	2.00435	(23.8446−31.7016)	2.06316	0.29779	(1.4795−2.6468)
BL	*c* = *k* = 0.6	27.67809	1.99749	(23.7630−31.5932)	2.04432	0.29507	(1.4745−2.6142)
BG		27.76365	2.00367	(23.8365−31.6908)	2.03969	0.29440	(1.4627−2.6167)
BL	*c* = *k* = −0.6	27.86918	2.01129	(23.9271−31.8113)	2.08318	0.30068	(1.4938−2.6725)
BG		27.77072	2.00418	(23.8425−31.6989)	2.05706	0.29691	(1.4751−2.6390)
BL	*c* = *k* = 1.4	27.57650	**1.99016**	**(23.6758−31.4772)**	2.02196	**0.29184**	**(1.4499−2.5940)**
BG		27.75895	2.00333	(23.8324−31.6855)	2.02902	0.29286	(1.4550−2.6030)
BL	*c* = *k* = −1.4	27.97448	2.01889	(24.0175−31.9315)	2.11054	0.30463	(1.5135−2.7076)
BG		27.77544	2.00452	(23.7043−31.7043)	2.06937	0.29869	(1.4839−2.6548)

ML: Maximum Likelihood, BG: General Entropy, BL: LINEX, BS: Squared Error, CI: Confidence/Credible Interval, s.e: Standard error.
